# Rhizosphere microbiome assemblage impacts soil chemical properties and medicinal quality in *Atractylodes chinensis*

**DOI:** 10.3389/fmicb.2026.1854479

**Published:** 2026-07-13

**Authors:** Yang Lu, Xiaoyan Cao, Chunhan Qiao, Yingzhe Li, Jia Bai, Tian Zhang, Yu Cao, Chunying Zhao

**Affiliations:** 1 Hebei Province Key Laboratory of Research and Development of Traditional Chinese Medicine, Institute of Chinese Materia Medica, Chengde Medical University, Chengde, Hebei, China; 2 State Key Laboratory for Quality Ensurance and Sustainable Use of Dao-di Herbs, National Resource Center for Chinese Materia Medica, China Academy of Chinese Medical Sciences, Beijing, China

**Keywords:** *Atractylodes chinensis*, atractylodin, microbial communities, rhizosphere, soil properties

## Abstract

**Introduction:**

Geographic variation markedly influences *Atractylodes chinensis* medicinal quality, driven by rhizosphere processes regulating atractylodin accumulation. Understanding soil-microbe-phytochemical interactions is essential for targeted cultivation to stabilize bioactive compound content.

**Methods:**

This study investigated the relationships among rhizosphere soil properties, microbial communities, and atractylodin content in *A. chinensis* from three geographic origins including high-content (HC; E118°48′55″, N41°21′40″), medium-content (MC; E115°31′30″, N40°29′36″), and low-content (LC; E117°40′22″, N41°20′24″). Analyses encompassed soil chemical properties (nutrients, trace elements), enzyme activities, and the composition of bacterial and fungal communities, alongside their correlations with atractylodin accumulation.

**Results:**

Significant inter-regional differences in soil properties were observed. MC soils exhibited the highest levels of organic matter, total nitrogen, and hydrolyzable nitrogen. LC soils contained elevated concentrations of trace elements (Zn, Cu, Fe, Mn) and total potassium. In contrast, HC soils possessed the highest magnesium content. Activities of soil phosphatases (alkaline phosphatase and neutral phosphatase) also differed significantly among sites. Microbial *α*-diversity peaked in MC soils. Distinct *β*-diversity patterns clearly differentiated the microbial communities of all three regions. In addition, bacterial community composition showed a stronger association with soil chemical parameters than fungal communities. The relative abundances of *Methylomirabilota* (bacteria) and *Mortierellomycota* (fungi) correlated positively with atractylodin content, whereas *Actinobacteriota* (bacteria) abundance correlated negatively.

**Discussion:**

These findings elucidate key ecological mechanisms driving variation in the medicinal quality of *A. chinensis* and provide practical insights for optimizing cultivation practices, including soil management and cultivar selection, to standardize quality and enhance stability of its medicinal value.

## 1 Introduction

Atractylodes refers to the dried rhizome of *Atractylodes lancea* (Thunb.) DC. or *Atractylodes chinensis* (DC.) Koidz., perennial herbaceous species within the Asteraceae family. Traditionally used in Chinese medicine, it is valued for various therapeutic effects (National Pharmacopoeia Commission, 2025). Modern pharmacological investigations have identified numerous bioactive constituents in *A. chinensis*, including atractylodin, atractylon, and *β*-eudesmol, which demonstrate significant anti-inflammatory, anti-tumor, and gastrointestinal regulatory activities (Zhan et al., 2024, Zhang et al., 2023, Zhu et al., 2018).

The accumulation of active ingredients in *A. chinensis* roots is critically influenced by the plant’s growing environment. Traditional Chinese Medicine (TCM) emphasizes the concept of Daodi (authentic medicinal herbs), positing that superior quality and efficacy stem from specific geographical and ecological conditions (Li et al., 2017). For *A. chinensis*, the content of key active components, notably atractylodin, exhibits substantial variation across different geographic origins, underscoring the pivotal role of provenance in determining its medicinal quality (Jing et al., 2026, Zhan et al., 2022). The formation of Daodi characteristics in medicinal plants is a multifaceted process. It is shaped by genetic factors, soil environment, climate, and agricultural management practices (Khan et al., 2020). Within this context, the rhizosphere microenvironment encompasses dynamic interactions among soil physicochemical properties, microbial communities, and plant roots. These interactions play a central role in regulating plant growth and the accumulation of secondary metabolites (Bulgarelli et al., 2013).

Soil constitutes the fundamental substrate for plant growth (Das et al., 2016). Rhizosphere microorganisms, as integral components of the soil ecosystem, profoundly impact the growth, accumulation of active ingredients, and stress tolerance of medicinal plants, thereby shaping their quality (Qu et al., 2020). These microbes participate in essential processes such as nutrient cycling, enhancement of stress tolerance, and disease suppression (Mendes et al., 2013). For instance, specific rhizobacteria solubilize insoluble phosphates, increasing phosphorus bioavailability (Lambers, 2022). *Mycorrhizal* fungi, particularly *Arbuscular mycorrhizal*, establish symbiotic relationships with plant roots, augmenting nutrient acquisition (especially phosphorus) and bolstering tolerance to abiotic and biotic stresses (Smith and Read, 2008; Bahadur et al., 2019). The composition and functional attributes of rhizosphere microbial communities are modulated by plant species, genotype, developmental stage, soil type, and agricultural management (Edwards et al., 2015).

Soil chemical properties directly affect plant growth and development while simultaneously shaping the structure and function of rhizosphere microbial communities (Fierer, 2017; Tedersoo et al., 2014). For example, elevated nitrogen levels can promote nitrogen-fixing bacteria, whereas limited phosphorus availability favors phosphate-solubilizing microorganisms (Wang et al., 2018). Conversely, microbial metabolic activities can alter soil properties while decomposition of organic matter (OM) releases nutrients, and microbial secretion of organic acids or alkaline compounds modifies soil pH (Gavazov et al., 2018).

Soil enzymes, including alkaline phosphatase, neutral phosphatase, urease, and dehydrogenase, serve as critical indicators of soil biological activity (Daunoras et al., 2024). They facilitate nutrient cycling by catalyzing the transformation of organic compounds into plant-available forms (Ali et al., 2025). Alkaline phosphatase and neutral phosphatase, for example, hydrolyze organic phosphorus esters, thereby increasing phosphate availability in soil (C. Li et al., 2024). Enzyme activities are influenced by both soil chemical properties and the resident microbial communities (Liu et al., 2022).

This study stratified *A. chinensis* rhizosphere soils from three geographic origins according to atractylodin content and analyzed differences in their chemical properties, enzyme activities, and microbial communities. By investigating the correlations among rhizosphere microorganisms, soil components, and atractylodin levels, we aim to establish a theoretical foundation for optimizing *A. chinensis* cultivation practices and ensuring consistent medicinal quality.

## 2 Materials and methods

### 2.1 Sample collection

**Figure 1:**
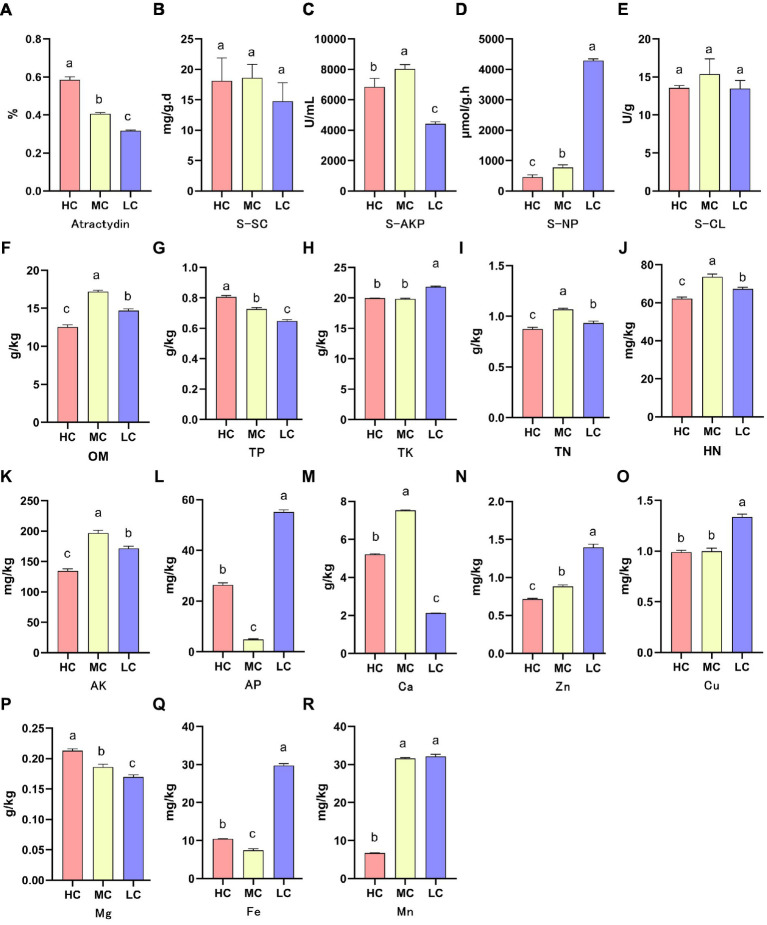
Content of atractylodin and various components in the rhizosphere soil (*n* = 3). **(A)** atractylodin content. **(B–E)** soil enzyme activity. **(F–R)** soil chemical composition content. The **(A–C)** in figures indicate significant difference (*p* < 0.05). The a, b, c in figures indicate significant difference (*p* < 0.05).

Following preliminary analysis of *A. chinensis* active ingredients across diverse geographic origins, rhizosphere soil samples were collected based on atractylodin content stratification: high-content (HC; Longitude E118°48′55″, Latitude N41°21′40″), medium-content (MC; E115°31′30″, N40°29′36″), and low-content (LC; E117°40′22″, N41°20′24″) (Figure 1A). Uniformly grown 4-year-old *A. chinensis* plants were selected. At the harvest stage, three individual plants spaced at least 10 m apart were randomly chosen within each geographic origin. Rhizosphere soil (defined as soil adhering to the root surface within 0–5 cm) was collected separately from each of the three plants. Each soil sample was individually sieved through a 50-mesh sieve without mixing, and the three samples were used as three biological replicates. The samples were immediately placed in an ice box and transported to the laboratory. Samples were partitioned as one portion for chemical and enzymatic analyses, the other submitted to Shanghai Majorbio Bio-pharm Technology Co., Ltd. for microbial sequencing.

### 2.2 Atractylodin quantification

Atractylodin content was quantified via high-performance liquid chromatography (HPLC) according to the Pharmacopoeia of the People’s Republic of China (2025), using SunFire C18 column (4.6 mm × 250 mm, 5 μm). The mobile phase consisted of methanol (A)-water (D) with gradient elution (detailed program provided in supplementary material). Detection wavelength was 340 nm, flow rate was 1 mL·min^−1^, and injection volume was 10 μL.

### 2.3 Soil chemical analysis

Soil parameters including total nitrogen (TN), total phosphorus (TP), total potassium (TK), hydrolyzable nitrogen (HN), available phosphorus (AP), available potassium (AK), OM, copper (Cu), iron (Fe), calcium (Ca), manganese (Mn), zinc (Zn), and magnesium (Mg) were analyzed following established methods and reference standards (Table 1).

**Table 1:** Detection methods and accordance of chemical components.

Chemical components	Detection methods and accordance	Chemical components	Detection methods and accordance
OM	NY/T 1121.6–2006	TK	NY/T 87–1988
TN	NY/T 1121.24–2012 3.1	AK	NY/T 889–2004 3.1
HN	LY/T 1228–2015 4	Ca, Mg	NY/T 1121.13–2006 (pH ≤ 7.5)
TP	NY/T 88–1988	Fe, Mn, Zn, Cu	NY/T 890–1,004 7.3.1
AP	NY/T 1121.7–2014		

### 2.4 Soil enzyme activity assay

Activities of soil alkaline phosphatase (S-AKP), neutral phosphatase (S-NP), cellulase (S-CL), and sucrase (S-SC) were determined using commercial kits (Beijing Solarbio Science & Technology Co., Ltd.; BC0280, BC0460, BC0150, BC0240) per the manufacturer’s protocol.

### 2.5 Microbial DNA extraction and amplification

Total genomic DNA was extracted from all nine soil samples using the E. Z. N. A. Soil DNA Kit (Omega Bio-tek, USA). DNA quality and concentration were assessed by 1.0% agarose gel electrophoresis and NanoDrop2000 spectrophotometry (Thermo Scientific, USA) and stored at −80 °C. The V3-V4 region of the bacterial 16S rRNA gene was amplified with primers 338F (5’-ACTCCTACGGGAGGCAGCAG-3’) and 806R (5’-GGACTACHVGGGTWTCTAAT-3’), and the fungal ITS region with primers ITS1F (5’-CTTGGTCATTTAGAGGAAGTAA-3’) and ITS2R (5’-GCTGCGTTCTTCATCGATGC-3’). PCR conditions: 95 °C for 3 min; 35 cycles of 95 °C for 30 s, 55 °C for 30 s, 72 °C for 45 s; final extension at 72 °C for 10 min (Bai et al., 2025).

### 2.6 Illumina sequencing and bioinformatic processing

Purified amplicons were pooled equimolarly and sequenced on an Illumina Nextseq2000 platform (Majorbio Bio-Pharm Technology Co. Ltd., China) using paired-end chemistry. Raw FASTQ files were demultiplexed, quality-filtered (fastp v0.19.6), and merged (FLASH v1.2.7). Taxonomy of operational taxonomic units (OTUs) were assigned using the RDP Classifier (v2.2). Functional prediction of bacterial OTUs was carried out using PICRUSt2, and Clusters of Orthologous Groups (COG) categories were annotated using the COG database.

### 2.7 Statistical analysis

Microbial community analysis was performed on the Majorbio Cloud platform (https://cloud.majorbio.com). Alpha diversity indices (Observed OTUs, Chao, Shannon, Good’s coverage) were calculated (Mothur v1.30.1). The similarity among the microbial communities in different samples was assessed via Principal Coordinate Analysis (PCoA) based on Bray–Curtis dissimilarity (Vegan v2.5–3). Statistical significance of group differences was evaluated using PERMANOVA (Adonis). Differentially abundant taxa (phylum to genus) were identified via LEfSe (*p* < 0.05). Due to multicollinearity among soil variables (VIF assessed using the car package), distance-based redundancy analysis (db-RDA; Vegan) with forward selection (Monte Carlo permutation, 9,999 permutations) determined key chemical drivers of bacterial community structure. Robust correlations were defined as Spearman’s |*ρ*| > 0.6 with *p* < 0.01. For the atractylodin, soil chemistry, and COG functional pathway analyses, differences among groups were evaluated using one-way analysis of variance (ANOVA) followed by the least significant difference (LSD) post-hoc test, performed with SPSS 25 (IBM, USA). Error bars indicate the standard error of the mean (SEM).

## 3 Results

### 3.1 Rhizosphere soil chemical composition and enzyme activities

Activities of S-AKP and S-NP differed significantly (*p* < 0.05) across samples, whereas S-SC and S-CL showed no statistical differences (Figures 1B–E). Among 13 quantified chemical elements, LC exhibited significantly higher (*p* < 0.05) TK, AP, Zn, Cu, Fe, and Mn than HC and MC. MC contained the highest OM, TN, HN, AK, and Ca, while HC showed elevated Mg content (Figures 1F–R).

### 3.2 Distinct microbial diversity patterns

**Figure 2:**
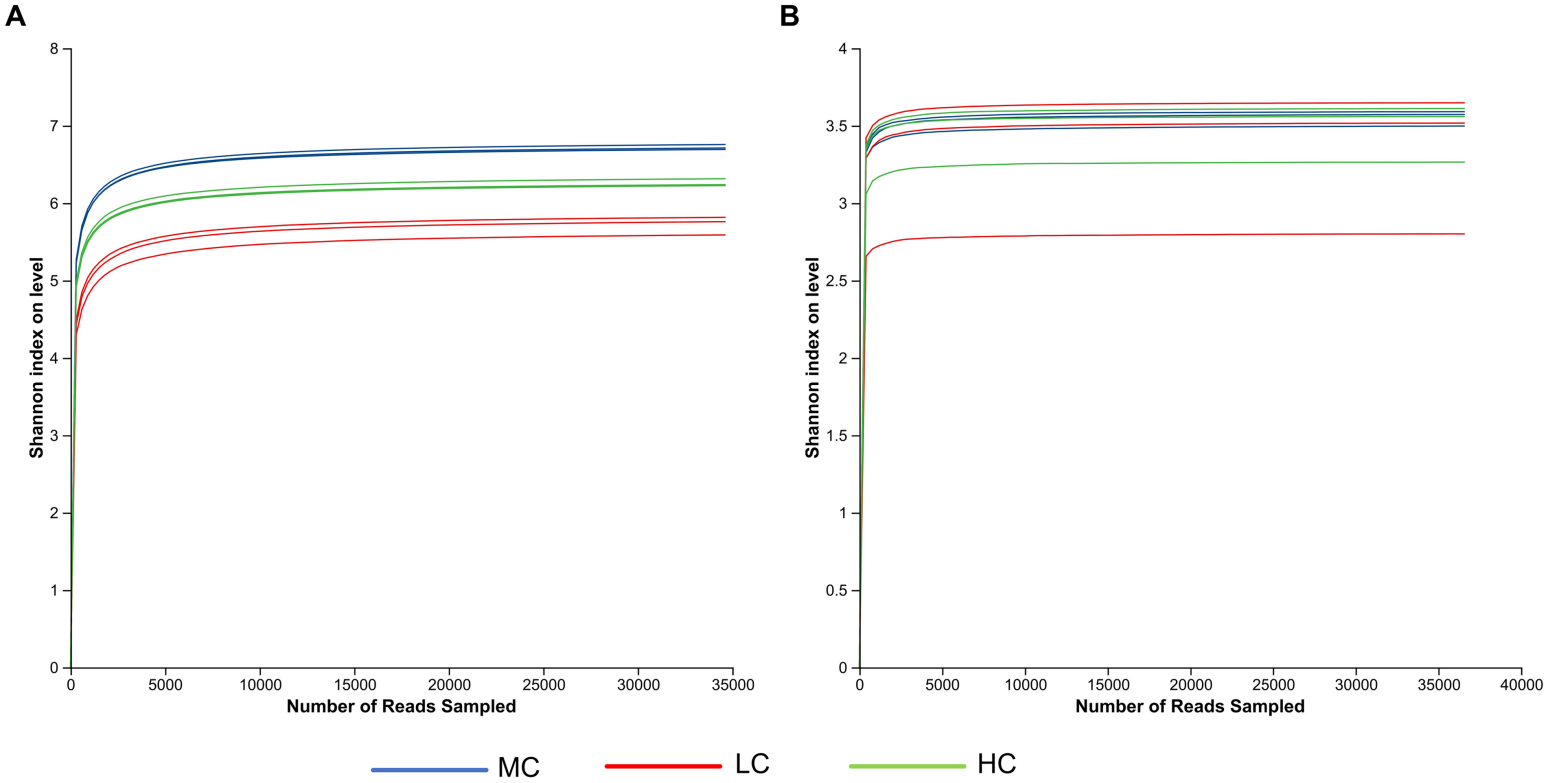
Rarefaction curve at the OTU level. **(A)** bacteria and **(B)** fungi.

**Figure 3:**
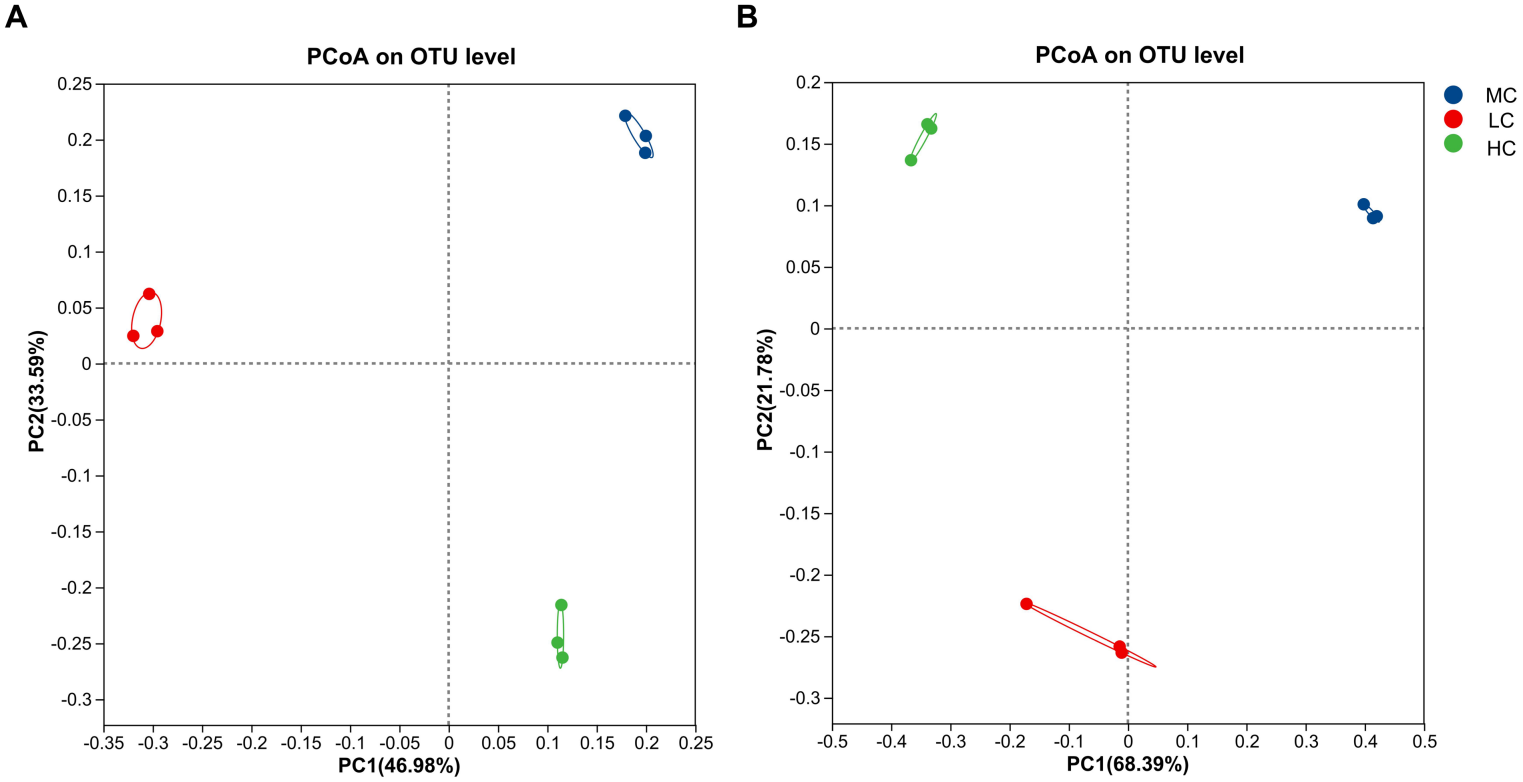
PCoA plot on OTU level of microbial communities. **(A)** bacteria and **(B)** fungi.

Rarefaction curves plateaued for all samples (Shannon index ordinate; Figure 2), confirming sufficient sequencing depth. Coverage indices indicated comprehensive microbial detection. Significant *α*-diversity differences (*p* < 0.05) were observed in bacterial and fungal communities (Chao, Sobs, Shannon, Simpson indices; Table 2), with MC exhibiting the highest diversity and richness. After processing 31,413 bacterial sequences (classified into 30 phyla, 428 genera, 1860 OTUs) and 38,377 fungal sequences (15 phyla, 167 genera, 277 OTUs), PCoA based on Bray–Curtis dissimilarity revealed distinct clustering among HC, MC, and LC groups for both bacterial and fungal communities (Figure 3). Adonis confirmed significant differences between groups for bacteria (R^2^ = 0.80271, *p* = 0.004) and for fungi (R^2^ = 0.88803, *p* = 0.004).

**Table 2:** Microbial diversity index of different rhizosphere soil samples.

Species	Diversity index	Samples		
		HC	MC	LC
Bacteria	Chao	5512.44 ± 266.01b	6539.54 ± 1002.08a	6264.52 ± 564.12a
	Sobs	3959.0 0 ± 32.51c	4772.67 ± 297.71a	4418.00 ± 138.69b
	Shannon	6.51 ± 0.04b	7.06 ± 0.02a	6.02 ± 0.11c
	Simpson	0.01 ± 0.00b	0.00 ± 0.00c	0.05 ± 0.00a
	Coverage	0.96 ± 0.00a	0.95 ± 0.01a	0.95 ± 0.01a
Fungi	Chao	512.96 ± 79.58c	643.47 ± 33.65a	566.41 ± 76.59b
	Sobs	439.33 ± 61.78b	533.33 ± 29.84a	451.67 ± 70.73b
	Shannon	3.52 ± 0.20fa	3.59 ± 0.05a	3.34 ± 0.47b
	Simpson	0.09 ± 0.03a	0.07 ± 0.00b	0.08 ± 0.04a
	Coverage	1.00 ± 0.00a	1.00 ± 0.00a	1.00 ± 0.00a

Values are means±standard deviations (*n* = 3), and a, b, c indicate significant difference (*p* < 0.05).

### 3.3 Microbial community composition and heterogeneity

**Figure 4:**
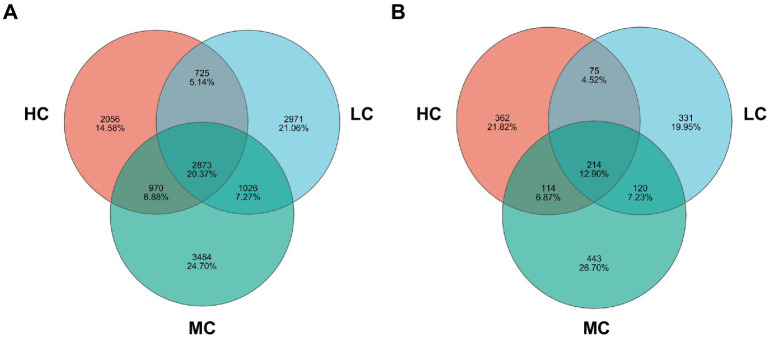
Comparison of OTU level. **(A)** bacteria and **(B)** fungi.

**Figure 5:**
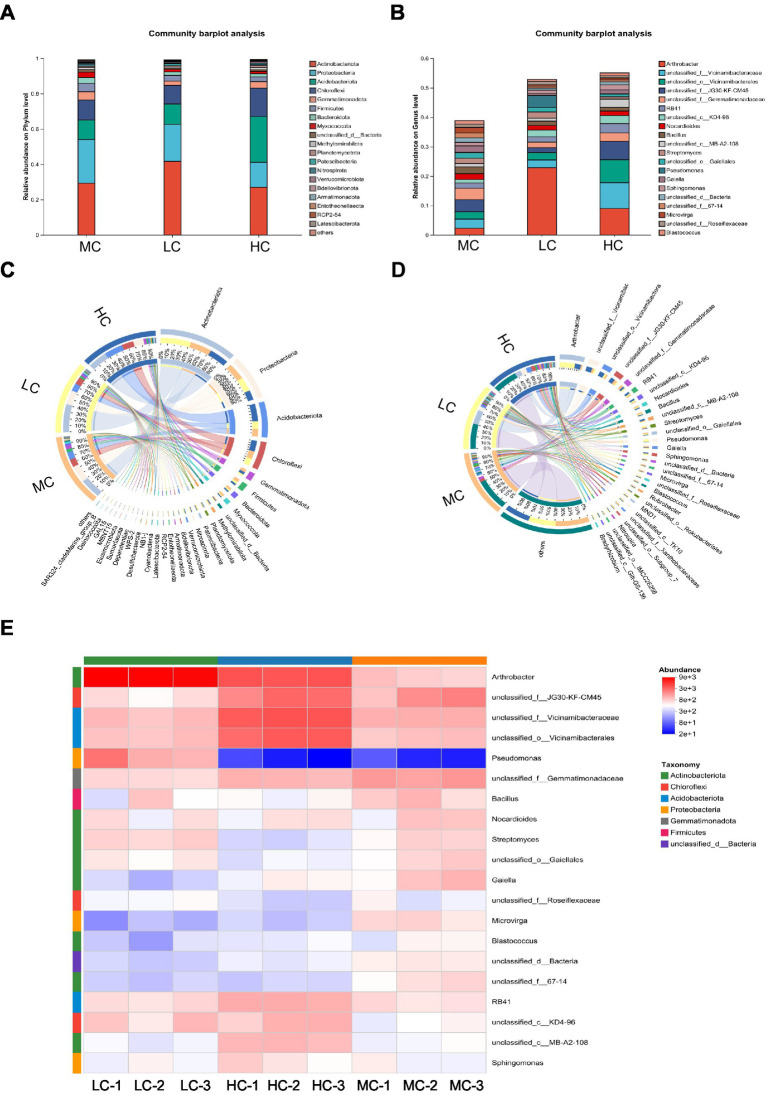
Bacteria community. **(A)** phylum level and **(B)** genus level. **(C,D)** Species proportion in samples. The left half of the Circos plot illustrates the species composition within each sample, right half of the plot shows the distribution proportion of species across different samples at the target taxonomic level. The outer ring indicates sample groups and the taxonomic classification of bacteria at phylum level **(C)** or genus level **(D)**, while the inner ring quantifies the relative abundance (percentage) of each taxon. **(E)** Differences in the relative abundance of genus across samples.

**Figure 6:**
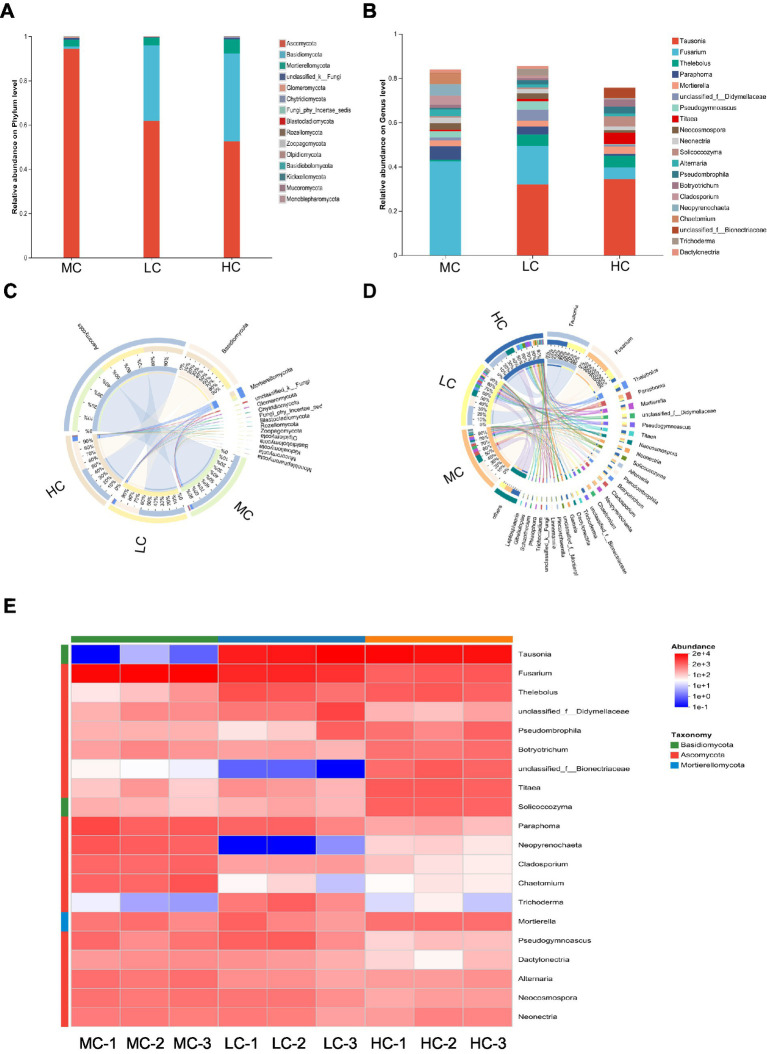
Fungi community. **(A)** phylum level and **(B)** genus level. **(C,D)** Species proportion in samples. The left half of the Circos plot illustrates the species composition within each sample, right half of the plot shows the distribution proportion of species across different samples at the target taxonomic level. The outer ring indicates sample groups and the taxonomic classification of fungi at phylum level **(C)** or genus level **(D)**, while the inner ring quantifies the relative abundance (percentage) of each taxon. **(E)** Differences in the relative abundance of genus across samples.

Shared OTUs comprised 2,873 bacterial and 214 fungal OTUs across all samples. Unique bacterial OTUs numbered 2,056 (HC), 3,484 (MC), and 2,971 (LC); fungal OTUs were 362 (HC), 443 (MC), and 331 (LC) (Figure 4). Dominant bacterial phyla included *Actinobacteriota* (27.02–41.71%), *Proteobacteria* (14.16–24.74%), and *Acidobacteriota* (11.05–25.92%), collectively encompassing all 20 dominant genera (Figures 5A–D). Genera *Arthrobacter*, *unclassified_f_JG30-KF-CM45*, *unclassified_f_Vicinamibacteraceae*, *unclassified_o_Vicinamibacterales* and *Pseudomonas* exhibited significant inter-group variation (Figure 5E). Fungal communities were dominated by *Ascomycota* (52.50–94.30%), *Basidiomycota* (1.13–39.81%), and *Mortierellomycota* (3.03–6.33%) (Figures 6A,C). All top 20 fungal genera including *Tausonia*, *Fusarium*, *Thelebolus*, *Paraphoma*, *Mortierella*, *unclassified_f_Didymellaceae*, *Pseudogymnoascus*, *Titaea*, *Neocosmospora*, *Neonectria*, *Solicoccozyma*, *Alternaria*, *Pseudombrophila*, *Botryotrichum*, *Cladosporium*, *Neopyrenochaeta*, *Chaetomium*, *unclassified_f_Bionectriaceae*, *Trichoderma* and *Dactylonectria* showed significant abundance differences (*p* < 0.05; Figures 6B,E).

### 3.4 Bacterial metabolic pathway shifts

**Figure 7:**
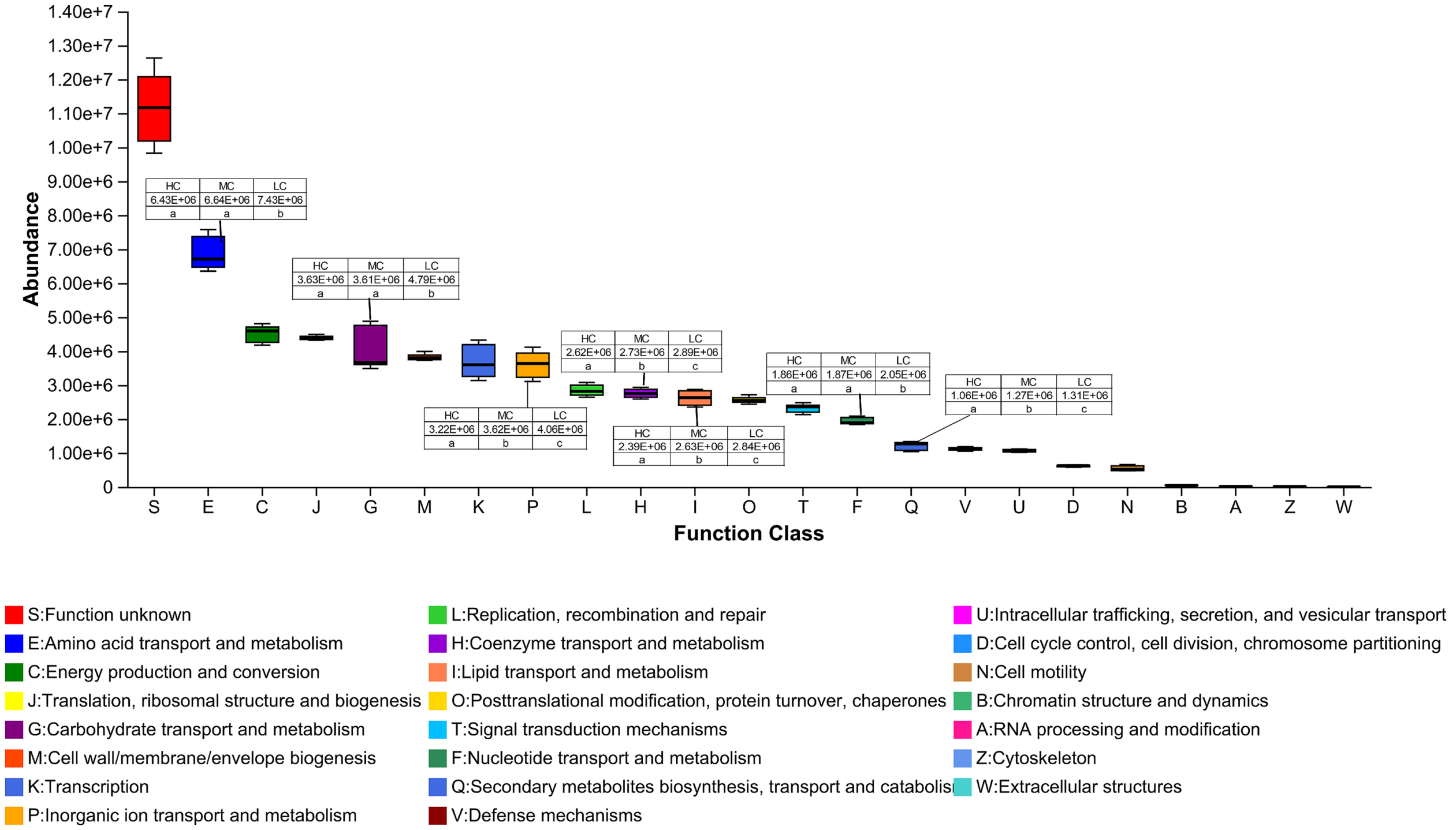
Clusters of Orthologous Groups function classification. Tables on figure present the microbial communities abundance of samples across various metabolic pathways, and a, b, c indicate significant difference (*p* < 0.05).

Functional annotation of bacterial OTUs identified 23 COG categories. Seven key metabolic pathways including Amino acid transport and metabolism, Nucleotide transport and metabolism, Carbohydrate transport and metabolism, Coenzyme transport and metabolism, Lipid transport and metabolism, Inorganic ion transport and metabolism, Secondary metabolites biosynthesis, transport and catabolism exhibited significant abundance variations across HC, MC, and LC (*p* < 0.05; Figure 7).

### 3.5 Microbiome-environment correlations

**Figure 8:**
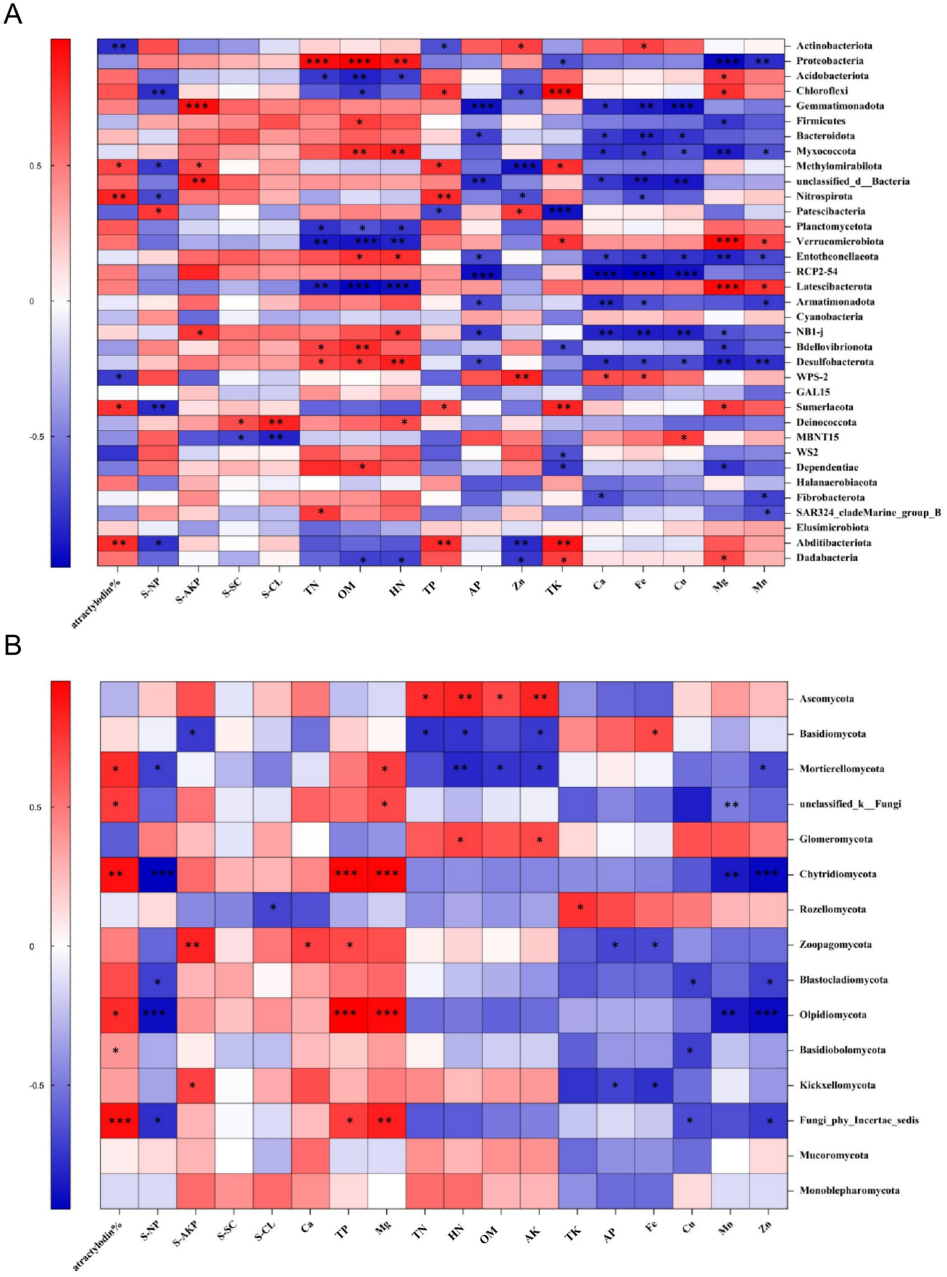
Heatmap of correlation analysis, abscissa represents atractylodin and soil chemical components, ordinate represents phylum of bacteria **(A)** and fungi **(B)**. Red indicates a positive correlation, and blue indicates a negative correlation. *, **, and *** are *p* < 0.05, *p* < 0.01, and *p* < 0.001, respectively.

Correlation analysis revealed stronger associations between bacterial phyla and soil chemistry than fungal phyla. Atractylodin content correlated negatively with *Actinobacteriota* and *WPS-2*, but positively with bacteria *Methylomirabilota*, *Nitrospirota*, *Sumerlaeota*, *Abditibacteriota*, and fungi *Mortierellomycota*, *unclassified_k_Fungi*, *Chytridiomycota*, *Olpidiomycota*, *Basidiobolomycota*, *Fungi_phy_Incerae_sedis*. Enzyme activities correlated with microbial taxa, negative S-NP and positive S-AKP. TN, HN, and OM collectively correlated negatively with *Acidobacteriota*, *Planctomycetota*, *Verrucomicrobiota* and *Latescibacterota*, but positively with *Proteobacteria* and *Desulfobacterota* (Figure 8A). Trace elements (Ca, Fe, Cu) showed negative correlations with eight bacterial phyla. In terms of fungal, TN, HN, OM, and AK showed positive correlations with *Ascomycota*, through TP and Mg correlated negatively with *Chytridiomycota*, *Olpidiomycota* and *Fungi_phy_Incertae_sedis* (Figure 8B).

## 4 Discussion

This study delineated systematic linkages among rhizosphere microbial communities, soil chemical properties, and atractylodin accumulation in *A. chinensis* across three geographically distinct producing areas. Our findings revealed complex soil-microbe-phytochemical interactions governing medicinal quality variation, providing ecological insights for cultivation optimization.

### 4.1 Soil chemistry as a microbial determinant

The pronounced heterogeneity in soil chemistry among HC, MC, and LC groups substantiates their function as primary environmental filters governing rhizosphere microbial assembly. MC soils distinguished by elevated OM, TN, and HN supported maximal microbial *α*-diversity. This aligns with the established edaphic principle wherein enhanced carbon and nitrogen availability facilitates microbial niche partitioning through resource provisioning (Shu et al., 2023, Y. Gu et al., 2023). Conversely, LC soils exhibited increased trace elements (Zn, Cu, Fe, Mn) and TK, imposing metal-mediated selection pressure via enzymatic inhibition and membrane destabilization in sensitive taxa (Yang et al., 2015; Iven et al., 2022). Abundant OM/TN/HN in MC promotes primary growth at the expense of secondary metabolism, reducing atractylodin allocation (Iqbal et al., 2025). Magnesium emerged as a pivotal regulatory element, demonstrating inverse correlation patterns relative to TN/HN/OM. As an essential cofactor for ATP-dependent enzymes and cell wall biosynthesis (Zhao et al., 2020), Mg attained peak concentrations in HC soils and correlated inversely with dominant phyla including Actinobacteriota. This implicates Mg-dependent regulation of community structure through specialized metabolic pathways, consistent with documented Mg-induced shifts in nutrient-cycling taxa (Andreini et al., 2008). Collectively, these chemical gradients underpin the observed *β*-diversity segregation (PCoA), confirming soil matrix heterogeneity as a fundamental driver of rhizosphere microbial biogeography (Gao et al., 2022).

### 4.2 Microbial mediators of atractylodin accumulation

Bacterial communities demonstrated significantly stronger correlations with atractylodin content than fungal communities, indicating their predominant role in regulating this key secondary metabolite. *Actinobacteriota* acts as key players in the turnover of particulate organic matter, particularly in detritusphere and later-stage rhizosphere habitats (Nuccio et al., 2021). The dominant phylum *Actinobacteriota* (27.02–41.71% relative abundance) exhibited a significant negative correlation with atractylodin accumulation. This inverse relationship aligns with observations in other medicinal plants where *Actinobacteriota* abundance negatively correlates with bioactive compound yields (Sepp et al., 2023). It is important to note that the correlations observed in this study are based on co-occurrence patterns and do not demonstrate causal or mechanistic relationships. Factors such as unmeasured environmental variables, indirect community interactions, or shared responses to soil properties could underlie the observed associations. Therefore, while our data suggest a potential link between *Actinobacteriota* abundance and atractylodin content, direct metabolism of this compound by *Actinobacteriota* has not been demonstrated, and experimental approaches (e.g., culture-based assays, isotopic tracing, or genetic manipulation) are required to test causality. Conversely, *Methylomirabilota* (bacteria) and *Mortierellomycota* (fungi) showed positive correlations with atractylodin. *Methylomirabilota* is specialized in methane oxidation and nitrogen cycling (Singh and Dubey, 2018). *Mortierellomycota*, a rhizosphere-abundant fungal phylum, potentially stimulates atractylodin biosynthesis through phosphorus solubilization and auxin production (Chen et al., 2025). These taxa represent candidate beneficial microbiome components for targeted bio-stimulant development. Genus-level analyses further revealed taxon-specific environmental responses, with *Pseudomonas* enriched in MC soils likely enhancing phosphorus availability via solubilization activity (Ettwig et al., 2010). Crucially, bacterial phyla exhibited significantly stronger associations with soil chemical parameters than fungal phyla (*p* < 0.01), consistent with their heightened sensitivity to edaphic factors including nutrient gradients and root exudate profiles.

### 4.3 Functional implications and metabolic trade-offs

Bacterial COG profiles revealed significant enrichment of carbohydrate and amino acid metabolism pathways in MC soils, providing functional evidence for microbial mediation of nutrient cycling. These pathways facilitate decomposition of complex OM into plant-available nutrients (Fan et al., 2025), supporting enhanced *A. chinensis* growth and secondary metabolite accumulation in MC. Conversely, the elevated abundance of secondary metabolite biosynthesis pathways in LC soils, despite reduced atractylodin content, implies either microbial competition for biochemical precursors or trace element inhibition of plant metabolic enzymes (Tang et al., 2022).

### 4.4 Enzyme activities as microbial functional proxies

Differential activities of soil phosphatases (S-AKP and S-NP) aligned with microbial functional profiles across sample groups. In MC soils, S-AKP activity demonstrated positive correlation with Proteobacteria abundance, likely enhancing phosphorus mineralization processes (Banerjee et al., 2018). Conversely, the negative association between S-NP and microbial communities in LC soils suggests impaired organic phosphorus decomposition under elevated trace element stress (Berendsen et al., 2012).

### 4.5 Practical implications for cultivation

These findings optimize strategies for enhancing *A. chinensis* medicinal quality. Actionable strategies involve organic amendments emulating MC-type soil conditions (balanced OM/TN/HN) sustain rhizosphere microbiome diversity and function (Gu et al., 2020), targeted inoculation with *Mortierellomycota* or *Methylomirabilota* consortia stimulates atractylodin biosynthesis, as evidenced in medicinal plant bioinoculation systems (Freedman et al., 2024), and mitigating excessive Zn/Cu/Fe levels prevents suppression of beneficial microbiota and enzymatic activity.

### 4.6 Limitations and future directions

One limitation of this study is that the three collection sites differed in multiple geographic and environmental attributes (e.g., altitude and likely climate conditions), which may also affect soil microbial community structure and plant metabolite profiles. While our analyses focused on soil properties and microbial composition, we cannot rule out the influence of unmeasured factors such as temperature, precipitation, and light intensity. Future studies incorporating long-term climatic monitoring and multi-site transplant experiments are needed to disentangle the relative contributions of soil biota versus broader environmental drivers. In addition, another limitation of this study is the relatively small number of biological replicates (*n* = 3 per site), which may not fully capture the natural spatial heterogeneity of soil microbial communities. Although three replicates are commonly used in soil microbial sequencing studies (Ahmad et al., 2023; Venturini et al., 2022), future investigations with larger sample sizes are needed to confirm the observed patterns.

## 5 Conclusion

This study established that atractylodin accumulation in *A. chinensis* was intrinsically linked to rhizosphere soil properties and microbial community composition across distinct geographic origins. Significant heterogeneity in soil chemical profiles, enzyme activities, and microbial community structures was observed among HC, MC, and LC groups. Bacterial taxa (notably *Actinobacteriota* and *Methylomirabilota*) exhibited stronger correlations with soil chemistry and atractylodin levels than fungal communities. The fungal phylum *Mortierellomycota* also demonstrated positive associations with atractylodin, indicating its potential role in facilitating secondary metabolite biosynthesis. Soil phosphatases (S-AKP and S-NP) functioned as critical regulators of nutrient cycling, modulating both microbial functionality and plant metabolic processes. These findings elucidate the mechanistic interplay between the rhizosphere environment and medicinal quality, providing a theoretical basis for optimizing cultivation through soil nutrient management and targeted microbial inoculation to enhance atractylodin yield and standardize *A. chinensis* production.

## Data Availability

The raw sequencing data have been deposited in the China National GeneBank Sequence Archive (CNSA) under accession number CNP0009564 (https://db.cngb.org/data_resources/project/CNP0009564/).
